# Supplementary material for: Printed Directional Bending Sensor with High Sensitivity and Low Hysteresis for Human Motion Detection and Soft Robotic Perception
Source: Sensors (Basel). 2023 May 24;23(11):5041. doi: 10.3390/s23115041 (PMC10255501; doi:10.3390/s23115041)
Supplement: Supplementary file 1 [file sensors-23-05041-s001.zip › Supporting information.pdf]

Supporting Information

Printed Directional Bending Sensor with High Sensitivity and Low Hysteresis for Human Motion Detection and Soft Robotic Perception

*Yi-Fei Wang *, Ayako Yoshida, Yasunori Takeda, Tomohito Sekine, Daisuke Kumaki, Shizuo Tokito **

Research Center for Organic Electronics (ROEL), Yamagata University, 4-3-16, Jonan, Yonezawa, Yamagata, 992-8510 Japan

Email: wang@yz.yamagata-u.ac.jp (Yi-Fei Wang)
tokito@yz.yamagata-u.ac.jp (Shizuo Tokito)

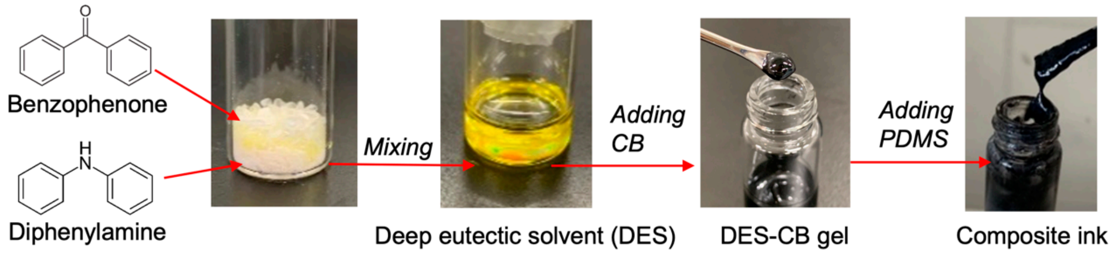

Figure S1. Illustration of the ink preparation process.

Table S1. The formulations of the printed inks. The amount of carbon black was calculated based on its weight percentage with PDMS.

Inks	***CB***	***PDMS***	***DES***
Porous composite CB-2wt%	*20 mg*	*980 mg*	*490 mg*
Porous composite CB-4wt%	*40 mg*	*960 mg*	*480 mg*
Random composite CB-5wt%	*50 mg*	*950 mg*	*0 mg*

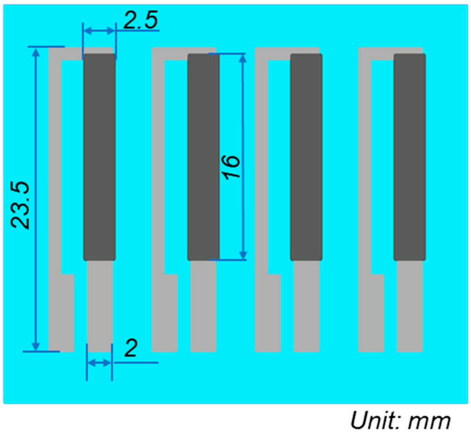

Figure S2. Geometry of printed sensors

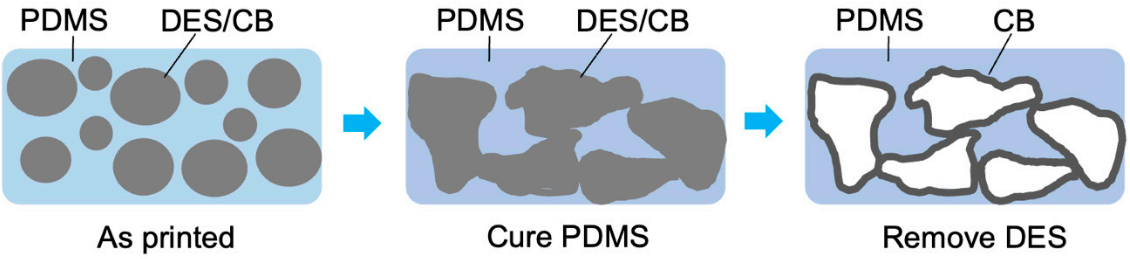

Figure S3. Illustration of the formation of porous conductive composite.

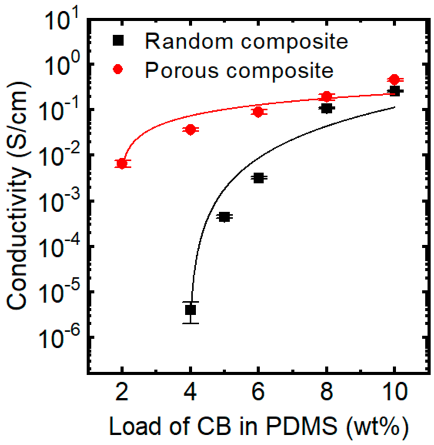

Figure S4. Conductivities of the printed composites with different loading of CB. In the random PDMS/CB composites, the conductivity and filler loading can be adapted by a power law, which offers a percolation threshold of ~3.94wt% loading of CB to PDMS. In the porous composites, the conductivity shows a relatively high value in all loading ratios with a percolation threshold of ~1.92wt% loading of CB to PDMS. Furthermore, with the same CB loading of 4wt%, the porous composite shows conductivity that is 4 orders of magnitude higher than that of the random PDMS/CB composite.

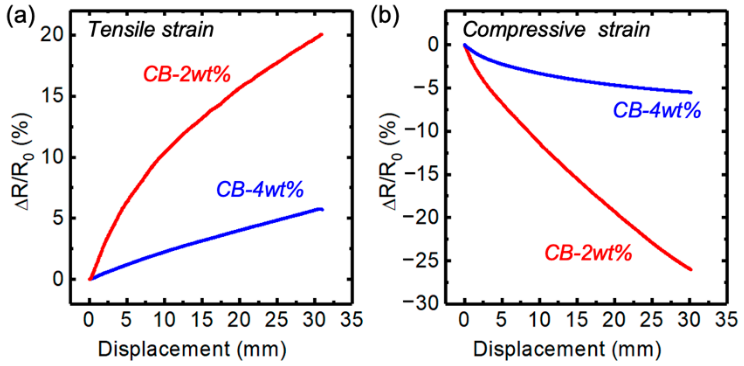

Figure S5. Bending sensing performance of porous composites with different CB loading. (a) Relative resistance changes of porous composite films with different CB loading under tensile strain. (b) Relative resistance changes of porous composite films with different CB loading under compressive strain.
